# A novel mouse model for liver metastasis of prostate cancer reveals dynamic tumour‐immune cell communication

**DOI:** 10.1111/cpr.13056

**Published:** 2021-05-21

**Authors:** Kaiyuan Liu, Na Jing, Deng Wang, Penghui Xu, Jinming Wang, Xinyu Chen, Chaping Cheng, Zhixiang Xin, Yuman He, Huifang Zhao, ZhongZhong Ji, Pengcheng Zhang, Wei‐Qiang Gao, Helen He Zhu, Kai Zhang

**Affiliations:** ^1^ Department of Urology State Key Laboratory of Oncogenes and Related Genes Renji‐Med‐X Stem Cell Research Center Ren Ji Hospital School of Medicine Shanghai Jiao Tong University Shanghai China; ^2^ School of Biomedical Engineering & Med‐X Research Institute Shanghai Jiao Tong University Shanghai China; ^3^ State Key Laboratory of Drug Research & Center of Pharmaceutics Shanghai Institute of Materia Medica Chinese Academy of Sciences Shanghai China

**Keywords:** liver metastasis, metastasis‐associated immune cell, metastatic microenvironment, prostate cancer

## Abstract

**Objectives:**

In contrast to extensive studies on bone metastasis in advanced prostate cancer (PCa), liver metastasis has been under‐researched so far. In order to decipher molecular and cellular mechanisms underpinning liver metastasis of advanced PCa, we develop a rapid and immune sufficient mouse model for liver metastasis of PCa via orthotopic injection of organoids from PbCre^+^; *rb1^f/f^;p53^f/f^* mice.

**Materials and Methods:**

PbCre^+^;*rb1^f/f^;p53^f/f^* and PbCre^+^;*pten^f/f^;p53^f/f^* mice were used to generate PCa organoid cultures in vitro. Immune sufficient liver metastasis models were established via orthotopic transplantation of organoids into the prostate of C57BL/6 mice. Immunofluorescent and immunohistochemical staining were performed to characterize the lineage profile in primary tumour and organoid‐derived tumour (ODT). The growth of niche‐labelling reporter infected ODT can be visualized by bioluminescent imaging system. Immune cells that communicated with tumour cells in the liver metastatic niche were determined by flow cytometry.

**Results:**

A PCa liver metastasis model with full penetrance is established in immune‐intact mouse. This model reconstitutes the histological and lineage features of original tumours and reveals dynamic tumour‐immune cell communication in liver metastatic foci. Our results suggest that a lack of CD8^+^ T cell and an enrichment of CD163^+^ M2‐like macrophage as well as PD1^+^CD4^+^ T cell contribute to an immuno‐suppressive microenvironment of PCa liver metastasis.

**Conclusions:**

Our model can be served as a reliable tool for analysis of the molecular pathogenesis and tumour‐immune cell crosstalk in liver metastasis of PCa, and might be used as a valuable *in vivo* model for therapy development.

## Graphical abstract

Liu et al. developed a rapid and immune sufficient mouse model for liver metastasis of prostate cancer via orthotopic injection of organoid cultures from PbCre^+^; *rb1^f/f^*;*p53^f/f^* mice. Using a niche‐labeling lentiviral reporter, a dynamic tumor‐immune cell communication at different phases in the liver metastatic niche is depicted. A paucity of CD8^+^ T cells and abundant PD1^+^ CD4^+^ T cells and CD163^+^ M2 macrophages contribute to an immune suppressive liver metastatic niche.

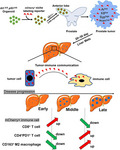

## 1 INTRODUCTION

Metastasis is the major cause of prostate cancer (PCa)‐related death.^1^ Bone metastasis, as one of the most common metastatic sites in advanced PCa, has been extensively investigated over the past decades.^2^ Accumulating evidence has demonstrated that liver is a frequently afflicted site in advanced PCa, especially in disease relapse after treatment of potent androgen receptor pathway inhibitors (ARPI).^3^ In particular, it has been reported by independent groups that a large portion of ARPI‐induced neuroendocrine prostate cancer (NEPC), an aggressive variant of castration‐resistant PCa, is associated with liver metastasis.^4^, ^5^ Currently, scarce treatment options such as chemotherapy for those patients often achieve poor responses. In spite of the daunting prognosis of PCa liver metastasis, the cellular and molecular mechanisms underlying PCa liver metastasis have been severely under‐researched so far.

Unlike bone metastasis that usually cause severe notable pain in PCa patients,^6^ liver metastasis is often symptomless at the beginning. Thus, when liver metastasis is clinically detected, many patients have reached the end stage of their disease.^7^ These clinical features make it difficult to collect liver metastatic samples from PCa patients for analysis. Current available genetically engineered mouse models (GEMM) or patient‐derived cell lines or xenografts fail to reconstitute the highly penetrant liver metastasis. Due to these long‐standing barriers and limitations, a stable, rapid and immune sufficient model of PCa liver metastasis is in urgent need to facilitate in‐depth mechanism investigations and new therapy development or drug screening.

When colonized in the liver, tumour cells encountered and communicated with local resident cells, especially immune cells, in the metastatic niche.^8^ A tumour supportive microenvironment in distal organs is required for the survival and growth of metastatic tumour cells.^9^ Although the ‘seed‐and‐soil’ theory can provide reasonable explanations for tumour metastasis,^10^ a comprehensive understanding of the dynamic composition of immune cells in the liver metastatic niche is still lacking. These unclear mechanisms that drive liver metastasis and the limited knowledge of tumour‐immune cell communication in PCa have highlighted the significance of an optimal *in vivo* model of liver metastatic PCa. In spite of a relatively low penetrance of distal metastasis, several lines of GEMM, such as PbCre^+^;*pten^f/f^;p53^f/f^* (*pten^Δ/Δ^p53^Δ/Δ^)*, PbCre^+^;*rb1^f/f^;p53^f/f^* (*rb1^Δ/Δ^p53^Δ/Δ^)* and PbCre^+^;*pten^f/f^;rb1^f/f^* (*pten^Δ/Δ^rb1^Δ/Δ^)* were currently used to study metastatic PCa.^11^, ^12^, ^13^, ^14^ These models had been well characterized by independent groups. For example, *pten^Δ/Δ^p53^Δ/Δ^* model revealed admixed adenocarcinoma and sarcomatoid histological feature, frequent lymph node metastasis and occasional lung metastasis.^11^ In contrast, *rb1^Δ/Δ^p53^Δ/Δ^* and *pten^Δ/Δ^rb1^Δ/Δ^* displayed prominent neuroendocrine differentiation and metastases in common sites including lymph node, lung, bone and liver.^12^, ^13^ Here, we utilize tumour organoids from *rb1^Δ/Δ^p53^Δ/Δ^* and *pten^Δ/Δ^p53^Δ/Δ^* GEMMs and generate a stable and rapid liver metastatic PCa mouse model with a high penetrance in immune‐sufficient mice. Using a mCherry^+^ niche‐labelling reporter system, dynamic tumour‐immune cell communications at different metastatic stage in the liver are depicted.

## 2 MATERIALS AND METHODS

### 2.1 Animals

All animal experiments in the current study were performed according to the ethical regulations of Renji Hospital. Animal experiment protocols were approved by the Renji Hospital Laboratory Animal Use and Care Committee. *Rb1^Δ/Δ^p53^Δ/Δ^* and *pten^Δ/Δ^p53^Δ/Δ^* mice were purchased from Jackson laboratory. C57BL/6 mice (male, 6‐8 weeks old) were purchased from Shanghai Slac Laboratory Animal Company. PCR Primers for *rb1^Δ/Δ^p53^Δ/Δ^* and *pten^Δ/Δ^p53^Δ/Δ^* mouse genotyping were provided in Supplemental Table 1.

### 2.2 Generation of murine prostate tumour organoids via magnetic cell sorting (MACS)

Murine prostate tumour tissues were collected, minced and digested in RPMI culture medium (A4192301, Gibco) containing 0.2 mg/mL collagenase I (17100017, Gibco), 0.2 mg/mL collagenase IV (17104019, Gibco), 0.01 mg/mL Dnase I (07900, STEMCELL Technologies), 0.1 mg/mL dispase (A002100‐0050, Sangon) and 2% FBS (10270‐106, Gibco). Digestion was performed on a shaker for 2 h at 37°C. Prostate tumour cells were blocked with the CD16/32 antibody for 40 minutes and stained in PBS with 2% FBS for 60 minutes on ice with EpCam antibody. Then, the anti‐Biotin MicroBeads (130‐090‐485, Miltenyi Biotec) were added into cell suspension and incubated for 15 minutes at 4℃. Cells were transferred to LS columns (130‐042‐401, Miltenyi Biotec) and passed through the magnetic field of a MACS Separator to isolate EpCam^+^ tumour epithelial cells. These purified tumour cells were then cultured in a previously reported method.^13^

### 2.3 Orthotopic inoculation in the murine prostate

MCherry^+^ niche‐labelling lentivirus infected organoids were injected into the anterior lobe of prostate of 6‐ or 8‐week‐old C57BL/6 mice with around 2,400 organoids for each mouse. Organoids were dissociated with 0.25% trypsin‐EDTA at 37°C for 1 minute. The organoid suspension was then mixed with Matrigel at 1:1 (v/v) ratio. Mice were anaesthetized using continuous flow of 1% isoflurane. The lower abdomen was sterilized with chlorhexidine iodine and a transverse incision of 1cm in length was made. 50 µL of organoid suspension was slowly injected into the anterior prostatic lobe where a bubble was clearly identified after inoculation. After inoculation, prostates were carefully put back into the body cavity, and the muscle and skin layer were then carefully sutured. Surgery was performed on a heating pad until mice completely recovered from anaesthesia.

Other detailed materials and protocols can be accessed in Supplemental Methods and Materials.

## 3 RESULTS

### 3.1 **Generation of two lines of tumour organoids from** *pten^Δ/Δ^p53^Δ/Δ^* and *rb1^Δ/Δ^p53^Δ/Δ^* **GEMMs**

**FIGURE 1:**
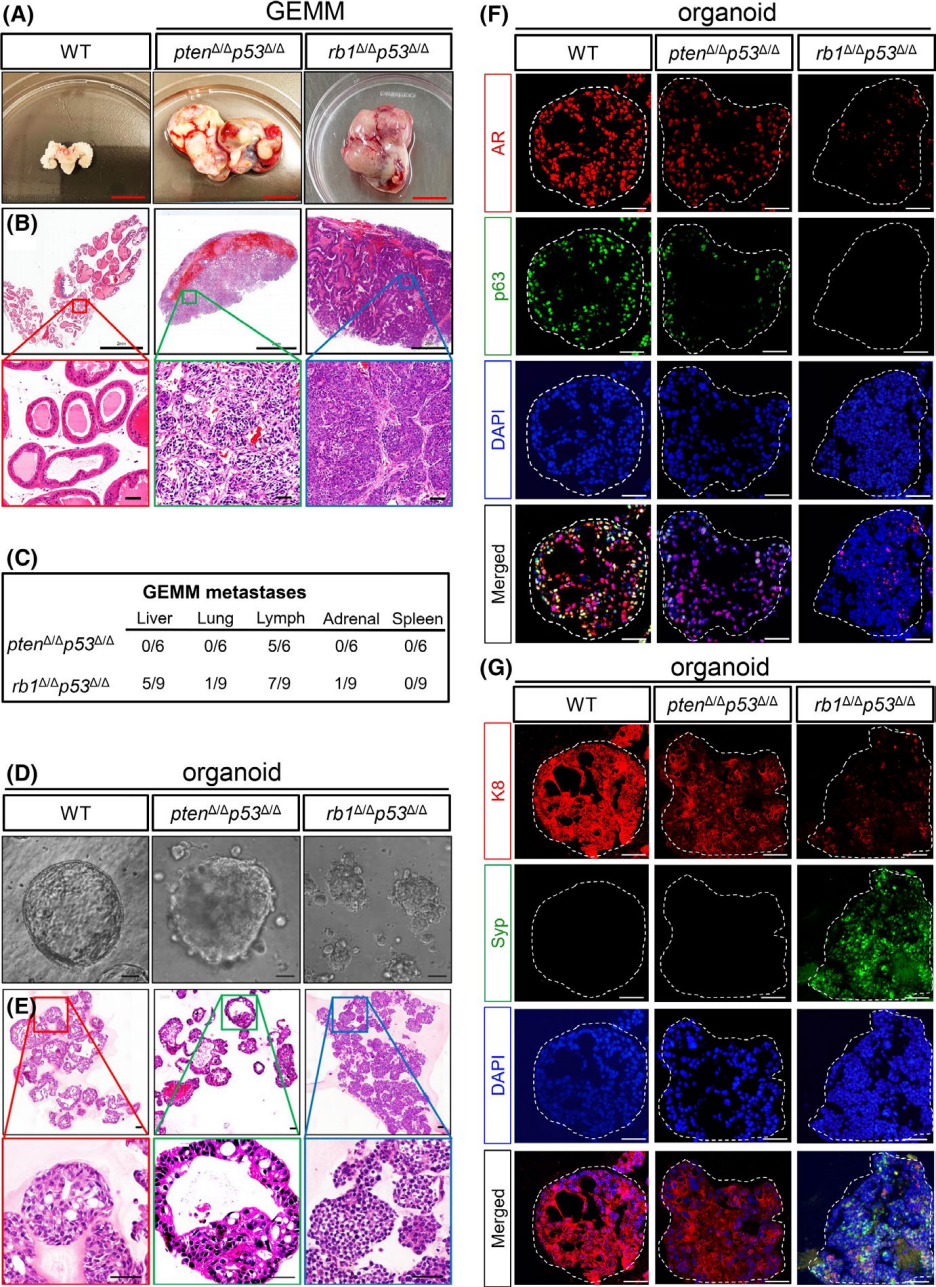
Generation of two lines of organoids from *pten^Δ/Δ^p53^Δ/Δ^* GEMM and *rb1^Δ/Δ^p53^Δ/Δ^* GEMM mice. A, Representative bright‐field images of the end stage prostate tumors from the *pten^Δ/Δ^p53^Δ/Δ^* GEMM (middle) and *rb1^Δ/Δ^p53^Δ/Δ^* GEMM (right) mice, respectively. An age‐matched prostate with seminal vesicles from a WT C57BL/6 mouse is shown as a control (left). Scale bar = 1 cm. B, Hematoxylin and eosin (H&E) staining of tissue sections from WT prostate, *pten^Δ/Δ^p53^Δ/Δ^* GEMM and *rb1^Δ/Δ^p53^Δ/Δ^* GEMM primary prostate tumors. Upper panel, scale bar = 2 mm. Lower panel, scale bar =50 μm. C, Quantifications of the penetrance of PCa metastasis in the *pten^Δ/Δ^p53^Δ/Δ^* GEMM (n = 6) and *rb1^Δ/Δ^p53^Δ/Δ^* GEMM (n = 9) mice. D, Phase contrast images of organoids from WT prostate (left), *pten^Δ/Δ^p53^Δ/Δ^* GEMM (middle) and *rb1^Δ/Δ^p53^Δ/Δ^* GEMM (right) prostate tumors. Scale bar = 50 μm. E, H&E staining of organoids from WT prostate (left), *pten^Δ/Δ^p53^Δ/Δ^* GEMM (middle) and *rb1^Δ/Δ^p53^Δ/Δ^* GEMM (right) prostate tumors, respectively. Scale bar =50 μm. F‐G, Coimmunofluorescent (IF) staining of AR and p63 (F), K8 and Syp (G) shows distinct lineage signatures of *pten^Δ/Δ^p53^Δ/Δ^* GEMM and *rb1^Δ/Δ^p53^Δ/Δ^* GEMM PCa organoids

Inactivation of *PTEN*, *TP53* and *RB1* is common driver genetic alteration in PCa.^15^ *Pten^Δ/Δ^p53^Δ/Δ^* and *rb1^Δ/Δ^p53^Δ/Δ^* GEMMs were utilized in this study. Then, we compared the median survival, primary tumours and overall metastases of these two mouse models. Genotypes of both mouse models were validated by PCR (Supplemental Figure 1A). These two mouse models shared a similar length of survival ranging from 28 weeks to 34 weeks. We euthanized the mice at around 30 weeks, when both mouse models reached the end stages of PCa. Typical images of primary tumours of *pten^Δ/Δ^p53^Δ/Δ^* (n = 6) and *rb1^Δ/Δ^p53^Δ/Δ^* (n = 9) GEMMs were shown in Figure 1A. Compared to *pten^Δ/Δ^p53^Δ/Δ^* GEMM tumours, the *rb1^Δ/Δ^p53^Δ/Δ^* PCa tissues displayed an evident hyperemic feature. In line with a previous study,^11^ adenocarcinoma and occasional squamous‐like tumours coexisted in the primary tumour of *pten^Δ/Δ^p53^Δ/Δ^* GEMMs. In contrast, *rb1^Δ/Δ^p53^Δ/Δ^* GEMM primary tumours were mainly featured with small‐cell like carcinoma (Figure 1B). These results indicate that different combinations of genetic deficiencies lead to discrepant histopathological presentations. We then examined the lineage signatures, including luminal cell maker K8 and neuroendocrine lineage gene Syp, on the primary tumours of both mouse models. Immunofluorescent (IF) staining showed that *pten^Δ/Δ^p53^Δ/Δ^* primary tumours expressed high level of K8 but rare Syp. In contrast, *rb1^Δ/Δ^p53^Δ/Δ^* GEMM primary tumours exhibited significant Syp expression but low level of K8. Therefore, compared to *pten^Δ/Δ^p53^Δ/Δ^* GEMM, *rb1^Δ/Δ^p53^Δ/Δ^* GEMM tumours displayed significant neuroendocrine features (Supplemental Figure 1B).

Next, we carefully assessed the distal metastasis in these two mouse models at the end stage. The major organs including the lymph node, liver, lung, spleen, adrenal gland, gastrointestinal tract, brain and bone were examined by microscopic inspection. As shown in Figure 1C, both mouse models exhibited frequent lymph node metastases. However, other metastases were rarely detected in *pten^Δ/Δ^p53^Δ/Δ^* GEMMs. In contrast, *rb1^Δ/Δ^p53^Δ/Δ^* GEMMs displayed prominent liver metastasis (5/9) and occasional lung (1/9) and adrenal gland metastases (1/9).

We then generated organoid lines from these two PCa models using a previously reported method,^16^ which allowed easy expansion and manipulation of organoids in vitro. The knockout efficiency of *pten*, *p53* and *rb1* in organoid lines were confirmed by qRT‐PCR after a long‐term culturing (Supplemental Figure 1C). As shown in Figure 1D,E, unlike the typical WT prostate organoids with round shape and regular sphere, *pten^Δ/Δ^p53^Δ/Δ^* tumour organoids exhibited malignant phenotypes including evident nuclear atypia, dense spheres and intruding pseudopodium. In contrast, *rb1^Δ/Δ^p53^Δ/Δ^* GEMM tumour organoids were featured with smaller cell size, with sheets or nest‐like histological structure. IF staining of androgen receptor (AR), basal cell maker p63, luminal cell marker K8 and neuroendocrine maker Syp were further performed in these two lines of organoids. As shown in Figure 1F,G, we found a coexistence of AR^+^ and p63^+^ tumour cells in most of *pten^Δ/Δ^p53^Δ/Δ^* GEMM tumour organoids (Figure 1F), but Syp^+^ cells were hardly detected. In contrast, *rb1^Δ/Δ^p53^Δ/Δ^* GEMM organoids contained a high percentage of Syp^+^ cells but expresses low levels of K8, AR and p63 (Figure 1G). We found that *rb1^Δ/Δ^p53^Δ/Δ^* GEMM organoids exhibited decreased expressions of *Ar* and *probasin* (*Pbsn*) but increased neuroendocrine marker *Chga* at mRNA levels (Supplemental Figure 1C). Collectively, these results suggest that the two lines of organoids in vitro stably sustain specific cell lineage signatures of their parental primary tumours *in vivo*.

### 3.2 *Rb1^Δ/Δ^p53^Δ/Δ^* organoid‐derived tumours (*rb1* ^Δ/Δ^ *p53* ^Δ/Δ^ ODT) recapitulate the histological and molecular features of the primary tumours

**FIGURE 2:**
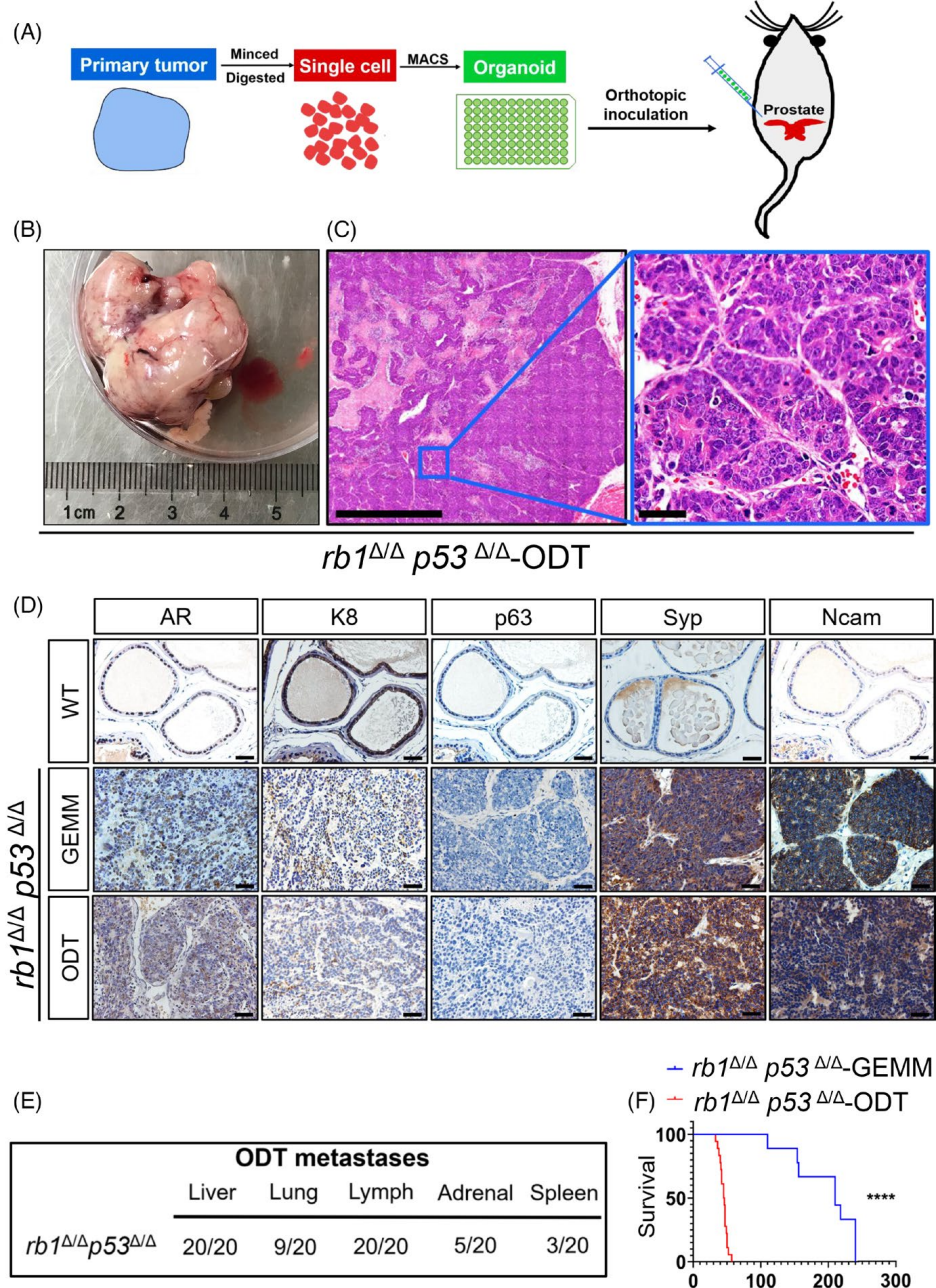
*Rb1^Δ/Δ^p53^Δ/Δ^* organoid derived tumors (*rb1^Δ/Δ^p53^Δ/Δ^* ODTs) recapitulate the histological features and lineage signatures of *rb1^Δ/Δ^p53^Δ/Δ^* GEMM primary PCas. A, Schematic illustration of experimental design to generate ODT. B, Representative bright‐field image of *rb1^Δ/Δ^p53^Δ/Δ^* ODT. C, H&E staining of the *rb1^Δ/Δ^p53^Δ/Δ^* ODT section. Scale bar = 50 μm. D, Immunohistochemistry (IHC) staining reveals the expression pattern of lineage markers in *rb1^Δ/Δ^p53^Δ/Δ^* GEMM primary tumor (the middle panel) and *rb1^Δ/Δ^p53^Δ/Δ^* ODT sections (lower panel). WT prostate sections are used as control (upper panel). Scale bar = 50 μm. E, Quantifications of the penetrance of PCa metastasis in the *rb1^Δ/Δ^p53^Δ/Δ^* GEMM organoid implanted mice (n = 20). F, Survival plot of *rb1^Δ/Δ^p53^Δ/Δ^* GEMM mice (n = 9) and *rb1^Δ/Δ^p53^Δ/Δ^* ODT bearing mice (n = 18). *P* < .0001, log rank

We then assessed the *in vivo* behaviour of the two lines of organoids (Figure 2A). A total of approximal 2,400 organoids was surgically inoculated into the anterior lobe of each prostate in WT C57BL/6 mouse. Mice implanted with *pten^Δ/Δ^p53^Δ/Δ^* organoids exhibited a relatively longer median survival of 60 days compared to 45 days for the mice implanted with *rb1^Δ/Δ^p53^Δ/Δ^* organoids. These *pten^Δ/Δ^p53^Δ/Δ^* and *rb1^Δ/Δ^p53^Δ/Δ^* organoid‐derived tumours (ODT) were collected, respectively, at around 40 days after inoculation (Figure 2B and Supplemental Figure 2A). H&E staining was performed to reveal the histological features of both *pten^Δ/Δ^p53^Δ/Δ^* (Supplemental Figure 2B) and *rb1^Δ/Δ^p53^Δ/Δ^* ODTs (Figure 2C). These two lines of ODT sections displayed very similar histological signature reminiscent of their respective primary tumours (Figures 1B, 2C and Supplemental Figure 2B). Of note, both AR^+^/K8^+^ adenocarcinoma and p63^+^/K5^+^ squamous‐like basal cell carcinoma were observed within a same section of *pten^Δ/Δ^p53^Δ/Δ^* ODT (Supplemental Figure 2C), in consistent with the primary tumour from *pten^Δ/Δ^p53^Δ/Δ^* GEMMs. Immunohistochemistry (IHC) staining suggested that *rb1^Δ/Δ^p53^Δ/Δ^* ODT expressed high level of neuroendocrine markers including Syp and Ncam. In contrast, luminal markers, such as AR and K8, and basal markers p63 were hardly detected in the *rb1^Δ/Δ^p53^Δ/Δ^* ODT section. These results suggested a similar histological feature and lineage signature between ODTs and their original GEMM primary tumours in both lines (Figure 2D).

### 3.3 *Rb1^Δ/Δ^p53^Δ/Δ^* ODT drives liver metastasis with full penetrance and high efficiency

Next, distal metastases were investigated in both *pten^Δ/Δ^p53^Δ/Δ^* and *rb1^Δ/Δ^p53^Δ/Δ^* organoid‐implanted mice. Similar to *pten^Δ/Δ^p53^Δ/Δ^* GEMMs, *pten^Δ/Δ^p53^Δ/Δ^* organoid‐implanted mice did not display significant distal metastases. In contrast, *rb1^Δ/Δ^p53^Δ/Δ^* organoid‐implanted mice rapidly developed distal metastases, in particular, a significant liver (20/20) metastasis with full penetrance (Figure 2E). Strikingly, *rb1^Δ/Δ^p53^Δ/Δ^* ODTs were capable to initiate liver metastasis as early as 20 ~ 30 days after implantation, compared to 28 weeks in *rb1^Δ/Δ^p53^Δ/Δ^* GEMM model for liver metastasis to be visible. Before the mice were succumbed, we found that the sizes of *rb1^Δ/Δ^p53^Δ/Δ^* ODT‐induced liver metastatic foci were significantly larger than those in the liver of *rb1^Δ/Δ^p53^Δ/Δ^* GEMMs (Supplemental Figure 3A,B). Moreover, the number of liver metastatic foci per lobe in *rb1^Δ/Δ^p53^Δ/Δ^* organoid‐implanted mice was dramatically higher than that of *rb1^Δ/Δ^p53^Δ/Δ^* GEMM counterparts (Supplemental Figure 3C). In order to exclude the possibility that orthotopic inoculation may introduce disruptions to the prostate gland or vasculature and lead to dissemination, fluorescent DiD‐labelled tumour cells were either intracardially or orthotopically injected in WT mice. The peripheral blood was collected from both groups at three time points after injections. As shown in Supplemental Figure 3D, DiD^+^ tumour cells were hardly detected in the peripheral blood of orthotopic injection group. However, significant amount of DiD^+^ tumour cells in the circulation were captured by flow cytometric analysis. Our results exclude the possibility of tumour cell leakage induced metastases during the implantation process.

Due to the massive liver metastasis, the liver weight of *rb1^Δ/Δ^p53^Δ/Δ^* organoid‐implanted mice were higher than normal livers from age‐matched mice (Supplemental Figure 3E). It's noteworthy that *rb1^Δ/Δ^p53^Δ/Δ^* organoid‐implanted mice had a significantly shorter median life span of 45 days and all died by 60 days after orthotopic inoculation, compared to 210 days of median life span in *rb1^Δ/Δ^p53^Δ/Δ^* GEMM (Figure 2F). We further compared the liver metastatic frequencies of intact organoids and single cell suspensions generated from fully digested organoids after orthotopic inoculation. As shown in Supplemental Figure 3F,G, those fully digested organoids showed significantly lower amount of liver metastasis as compared to intact organoids. These data suggested that intact cell‐cell communication in 3D organoid culture system was required for a high efficiency of liver metastasis. Moreover, we carefully evaluated the liver metastatic potential of these *rb1^Δ/Δ^p53^Δ/Δ^* organoids during long‐term culturing, freezing, thawing and serial passaging. No evident alterations were found in either the morphological feature (Supplemental Figure 3H) or metastatic penetrance *in vivo* of the organoids up to 20 serial passages. In summary, our results suggested that the *rb1^Δ/Δ^p53^Δ/Δ^* ODT model could be served as a reliable and fast model for liver metastasis of PCa.

**FIGURE 3:**
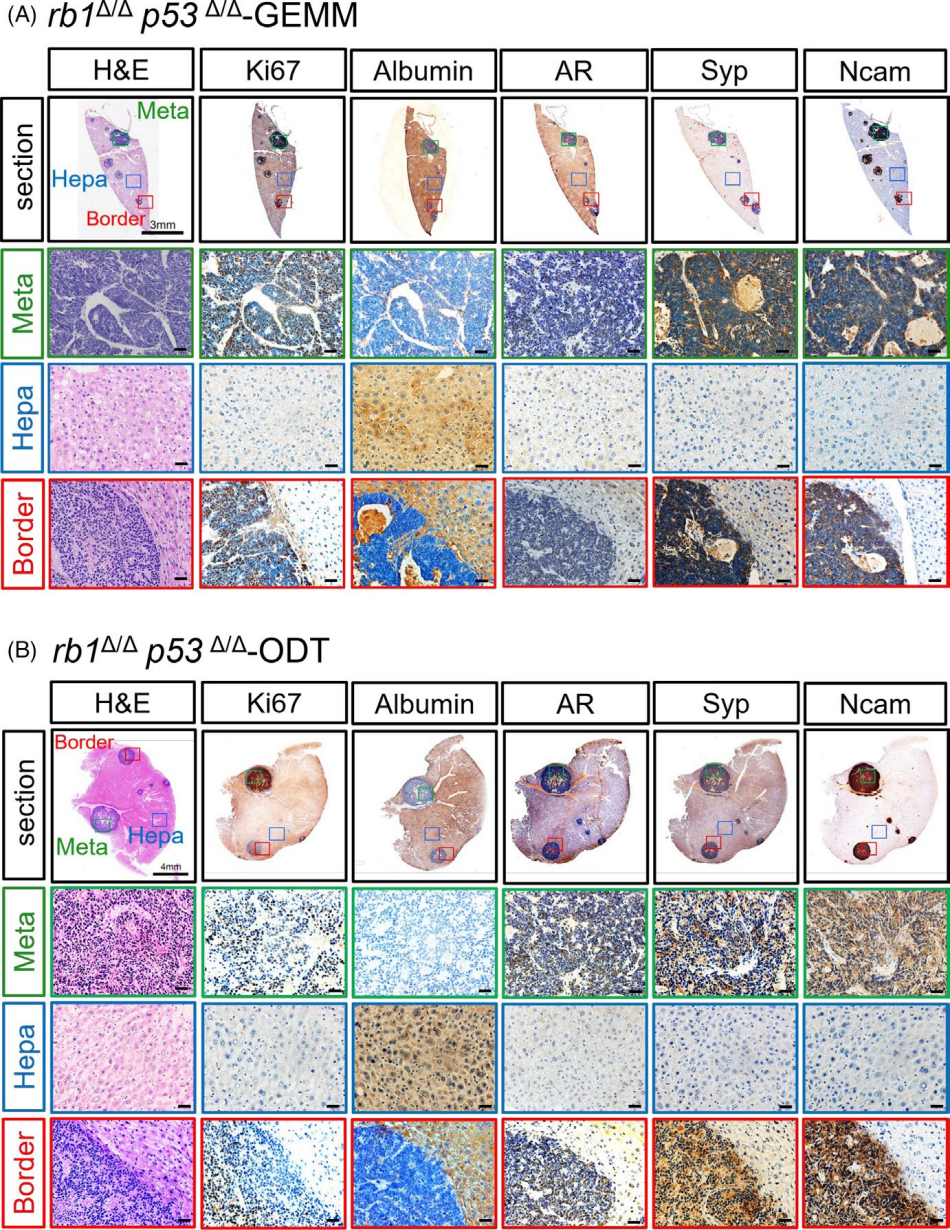
Establishment of a rapid liver metastasis model for PCa in immune‐sufficient mice using *rb1^Δ/Δ^p53^Δ/Δ^* GEMM organoids. A‐B, H&E and IHC staining images show the metastatic tumor foci in a liver lobe of *rb1^Δ/Δ^p53^Δ/Δ^* GEMM mice (A) and *rb1^Δ/Δ^p53^Δ/Δ^* GEMM organoids implanted mice (B). The metastatic region (meta, green), the border between the metastasis and the hepatocytes (border, blue) and the hepatic region (hepa, red) in the liver sections are shown. Ki67 antibody stains the proliferating cells of the tumor foci. Albumin is used as a marker of hepatocytes. AR antibody labels the tumor cells originated from the PCa. Syp and Ncam are used as markers for PCa cells with neuroendocrine differentiation phenotype

In order to validate whether the tumour foci in the liver are descendants of *rb1^Δ/Δ^p53^Δ/Δ^* GEMM primary tumour cells, H&E and IHC staining of paraffin embedded sections of livers from both *rb1^Δ/Δ^p53^Δ/Δ^* GEMM and *rb1^Δ/Δ^p53^Δ/Δ^* ODT mice were conducted. In both GEMM and ODT models, tumour cells in the metastatic foci displayed expression of Hoxb13 (Supplemental Figure 3I), low levels of AR and K8 but high levels of Syp and Ncam (Figure 3A,B), suggesting the prostatic origin and stable neuroendocrine signature of these metastatic tumour foci. Albumin, which was broadly expressed by hepatocytes, clearly distinguished the tumour cells and hepatocytes. Moreover, a high percentage in the tumour foci rather than the hepatocytes were stained positive for Ki67, reflecting an active proliferation status of tumour cells after they colonized in the liver. Collectively, these results showed that *rb1^Δ/Δ^p53^Δ/Δ^* ODT was capable to rapidly induce liver metastasis with a full penetrance. The metastatic tumour cells in the liver sustained a neuroendocrine feature and are highly proliferative.

### 3.4 A niche‐labelling system reveals tumour‐immune cell communications in liver metastatic niche

**FIGURE 4:**
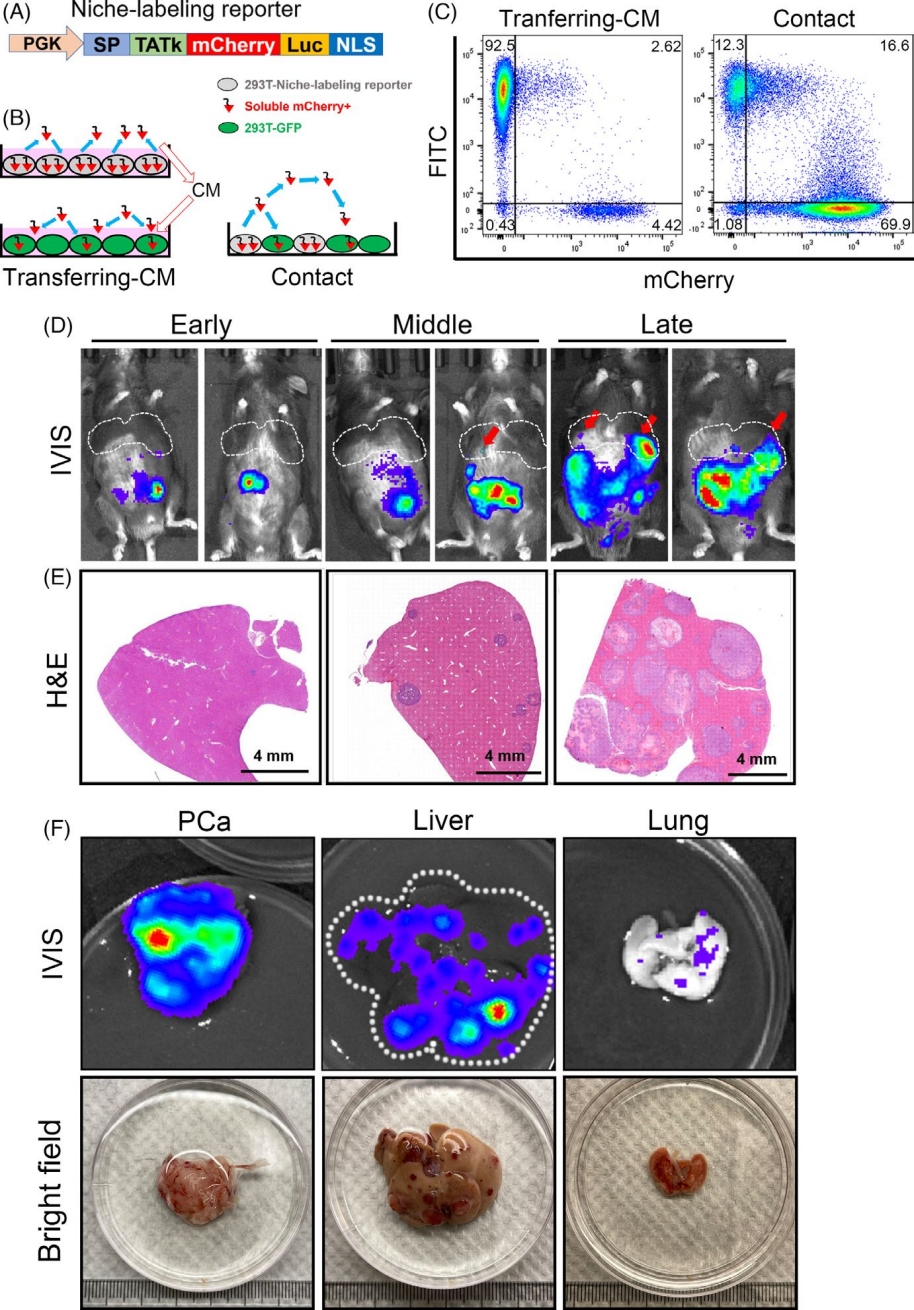
A niche‐labeling system to reveal tumor‐niche communications in the liver metastasis of *rb1^Δ/Δ^p53^Δ/Δ^* ODT mouse model. A, A lentiviral construction containing the PKG promoter driven transactivator of transcription (TATk) peptide followed by a secreted mCherry protein and a bioluminescent luciferase cassette. B, Proof of concept experiment of the niche labeling system using conditioned‐medium (CM) or directly contacted co‐culture method of 293T cells. C, Flow cytometry results show that the fluorescent mCherry protein can be secreted by mCherry^+^ 293T cells and uptaken by the GFP^+^ 293T cells. GFP^+^ 293T cells were cultured by transferring‐CM of mCherry^+^ 293T cells or directly co‐cultured with mCherry^+^ 293T cells in a contact manner. D, The bioluminescent images of *rb1^Δ/Δ^p53^Δ/Δ^* ODT mice at early (20 d after inoculation), middle (35 d after inoculation) and late stages (50 d after inoculation). The liver metastatic tumor foci are indicated by red arrows. *Rb1^Δ/Δ^p53^Δ/Δ^* GEMM organoids are infected with the niche‐labeling lentiviruses and orthotopically inoculated in the prostate of WT C57BL/6 mice (8‐weekold). E, H&E staining shows the liver metastatic foci in liver at early, middle, and late stages. Scale bar = 4 mm. F, The bioluminescent images and bright field images of PCa, liver and lung isolated from *rb1^Δ/Δ^p53^Δ/Δ^* ODT mice

When tumour cells colonized in the liver, they inevitably encountered with the local immune cells in the metastatic niche. In order to delineate the immune cell composition that responded to tumour cells in the liver metastasis, we generated a lentiviral niche‐labelling reporter that could both trace the *rb1^Δ/Δ^p53^Δ/Δ^* ODT cells and label their communicating immune cells in the microenvironment. A secreted mCherry protein with a lipid‐permeable transactivator of transcription (TATk) peptide as previously reported was fused with a luciferase reporter and cloned into a lentiviral vector (mCherry‐Luc reporter) (Figure 4A).^17^ Donor cells infected with the mCherry‐Luc reporter were capable to secrete soluble mCherry proteins which could be subsequently uptaken indiscriminately by neighbouring environmental cells. To validate this mCherry‐Luc reporter system in vitro, we infected 293T cells with the reporter lentivirus and GFP lentivirus, respectively (Figure 4B). The amount of sLP‐mCherry fluorescent protein ‘swallowed’ by GFP^+^ 293T were determined in a non‐contact or a contact manner using flow cytometric analysis. As shown in Figure 4C, GFP^+^ recipient 293T cells uptook significantly greater amount of mCherry proteins in a directly contacted co‐cultured system (16.6%) than that treated with conditioned‐medium (CM) (2.62%). These results suggested that cell‐cell contact contributed to a higher lipid permeable sLP‐mCherry uptake, and this mCherry‐Luc reporter was able to efficiently label niche cells.

Next, mCherry‐Luc reporter lentivirus infected *rb1^Δ/Δ^p53^Δ/Δ^* organoids were orthotopically inoculated in the prostate of WT C57BL/6 mice in order to directly study the cell communications between immune cells uptaking mCherry‐Luc and tumour cells. As shown in Figure 4D, the rapid outgrowth of *rb1^Δ/Δ^p53^Δ/Δ^* ODT in the prostate was clearly monitored by an IVIS bioluminescent imaging system. According to our observations, evident but weak bioluminescent signal in the lower abdomen could be detected at 20 ~ 30 days after orthotopic inoculation. Since liver metastasis could be detected by microscopic inspection at this stage (Figure 4E), we defined this period as the early stage. Around 30 ~ 40 days, bioluminescent intensity and H&E staining (Figure 4F) clearly showed the sporadic liver metastases at this stage (Figure 4E). We termed this period from 30 to 40 days as the middle stage. At 50 to 60 days, the intensity and region of bioluminescence expanded further with a prominent signal in the liver (pinpointed with red arrows). Accordingly, we termed this period from 50 to 60 days as the late stage of liver metastasis. As shown in Figure 4F, the primary prostate tumour, liver and lung from a *rb1^Δ/Δ^p53^Δ/Δ^* ODT implanted mouse at 50 days were isolated and detected positive for bioluminescent signal using IVIS system. The representative bright field and H&E staining images of tumour foci in the whole liver across different stages were shown in Supplemental Figure 4A. We also quantified the diameter of the metastatic tumour foci in the liver at different stages (Supplemental Figure 4B), with 20 μm at early stage, 200 μm at the middle stage, and over 2000 μm at the end stage (Supplemental Figure 4C). As luciferase and fluorescent proteins could be immunogenic *in vivo*, we performed experiments to compare tumour growth in the mCherry‐Luc transfected and non‐transfected ODT via subcutaneous or orthotopic injection. As shown in Supplemental Figure 4D, the growth kinetics of the subcutaneous tumours suggested that transfected tumours showed a delayed growth as compared to non‐transfected tumours. Considering the distinct subcutaneous and prostatic microenvironments, we also compared the growth rate of transfected and non‐transfected ODTs in the prostate. As shown in Supplemental Figure 4E, no significant difference in tumour weights was detected between these two groups. And no significant differences were found in immune cell infiltration including macrophages, neutrophils, B cells, CD4^+^ T cells and CD8^+^ T cells (Supplemental Figure 4F,G), suggesting that mCherry‐Luc niche‐labelling reporter did not introduce profound immunogenic effect at least in local prostatic microenvironment. Collectively, our results suggested that this niche‐labelling reporter was capable to vividly reflect both the status of primary tumour and the liver metastasis at different stages.

### 3.5 Depiction of the niche‐labelled immune cells in the liver metastasis of *rb1^Δ/Δ^p53^Δ/Δ^* ODT model

**FIGURE 5:**
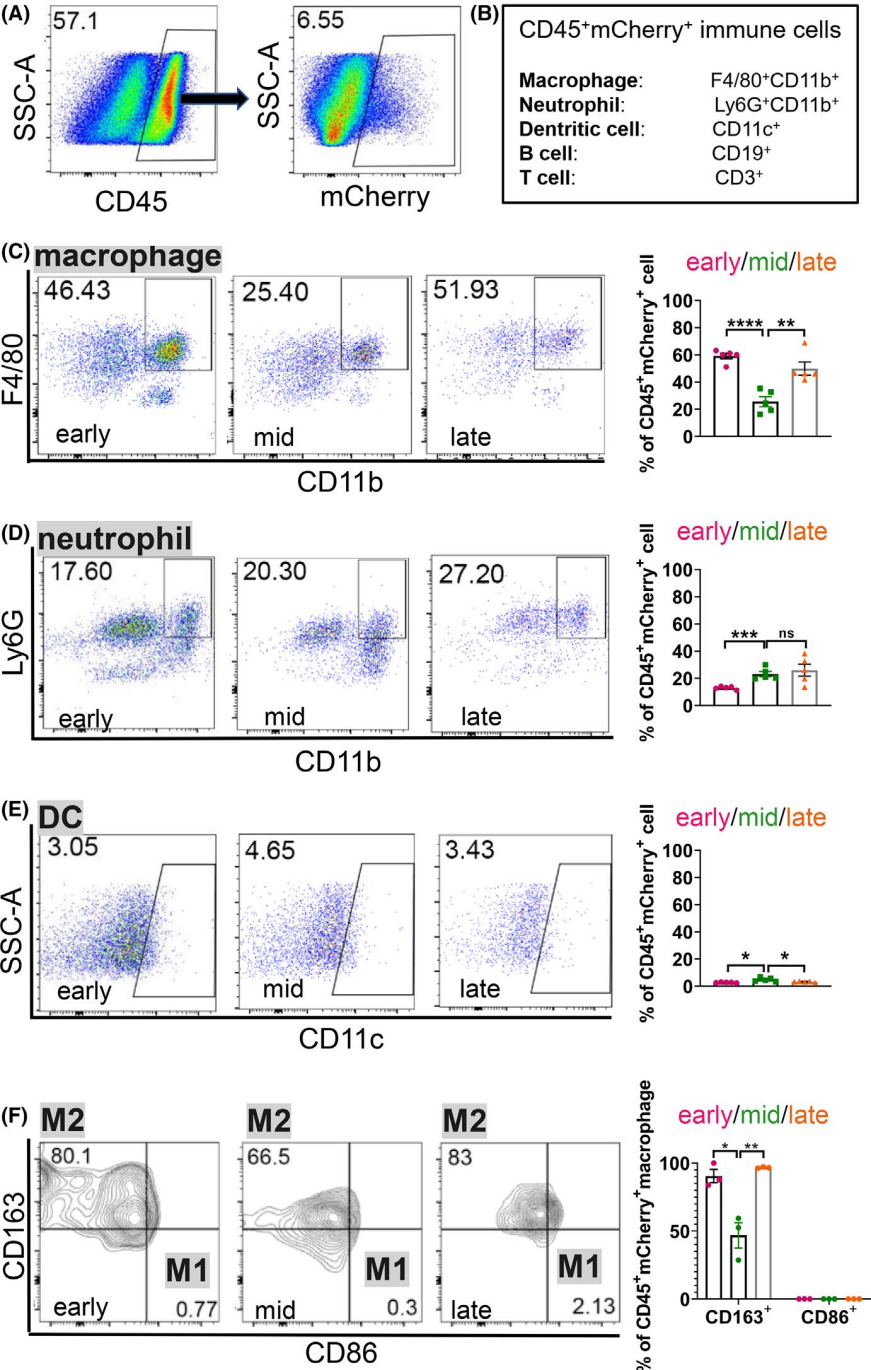
Immune‐suppressive CD163^+^ macrophages are significantly increased in the metastatic niche at the late stage of PCa liver metastasis. A, Flow cytometric analysis of immune cells that are mCherry^+^ in the liver metastasis of *rb1^Δ/Δ^p53^Δ/Δ^* ODT. B, A schematic of immune cells examined in the liver metastatic niche. C‐F, Representative flow cytometric plots and quantification of the ratio of macrophages (C), neutrophils (D) and dentritic cells (DCs) (E) in CD45^+^ mCherry^+^ cells, and M1, M2 macrophages (F) in CD45^+^ mCherry^+^ macrophages in *rb1^Δ/Δ^p53^Δ/Δ^* ODT liver metastasis niche at the early, middle and late stages. Unpaired two‐tailed Student's *t*‐test is used for statistical analysis. The data are presented as the means ± SEMs. *P < .05, **P < .01, ***P < .001, ****P < .0001, n = 5

In order to reveal the immune cell composition that uptook mCherry protein secreted by tumour cells in the liver metastatic niche, the liver was cut into pieces and generated into single cell suspension (Supplemental Figure 5A). Next, we analysed the mCherry and CD45 double positive (mCherry^+^ CD45^+^) cell population by flow cytometry (Figure 5A). Following the gating strategies in Supplemental Figure 5B,C, we deciphered the dynamic changes of macrophage (F4/80^+^ CD11b^+^), neutrophil (Ly6G^+^ CD11b^+^), dentritic cell (DC, CD11c^+^), B cell (CD19^+^) and T cell (CD3^+^) in mCherry^+^CD45^+^ niche‐labelled immune cells at early, middle and late stages of liver metastasis (Figure 5B). Macrophage (Figure 5C) and neutrophil (Figure 5D) were the most abundant fractions in tumour‐communicating immune cells in liver metastasis. Macrophages accounted for 46.43% of total CD45^+^ mCherry^+^ immune cells at the early stage, then dropped to 25.4% at the middle stage, and eventually rebounded to 51.93% at the late stage (Figure 5C). Distinct from macrophages, the ratio of neutrophils in CD45^+^ mCherry^+^ immune cells steadily increased from 17.6% at the early stage to 20.3% at the middle stage, then reached a peak value of 27.2% at the late stage (Figure 5D). Unlike macrophage and neutrophil, DC was relatively rare in mCherry labelled immune cells. As shown in Figure 5E, there was a slight increase in DC from early stage (3.05%) to middle stage (4.65%), then the ratio of DC dropped again (3.43%) as it came to the late stage.

It is well accepted that macrophage can be divided into M0, M1 and M2 polarized subsets.^18^ It is proposed that M2 macrophage displayed tumour‐permissive activity while M1 macrophage exhibited pro‐inflammatory functions.^19^ To characterize the phenotype of the mCherry^+^ macrophages at different stages in the metastatic niche, CD86 and CD163 were used to distinct M1 and M2 macrophage, respectively.^20^ As shown in Figure 5F, over 65% of macrophages were positive for CD163 expression, suggesting that M2 like macrophages accounted for the predominant subset in the mCherry^+^ macrophages. Moreover, the ratio of CD163^+^ M2 macrophage initially decreased from 80.1% at the early stage to 66.5% at the middle stage. Eventually, the ratio rebounded to 83% as it came to the late stage. Collectively, our results revealed that macrophage and neutrophil were the most sufficient immune cell subsets that interacted with invading tumour cells in the liver metastases. The predominant subset of M2‐like macrophages reached a peak value at the late stage, suggesting that an immune‐suppressive microenvironment was created in the liver metastatic niche.

### 3.6 A paucity of CD8^+^ T cells and abundant PD1^+^CD4^+^ T cells contribute to an immune‐suppressive liver metastatic niche

**FIGURE 6:**
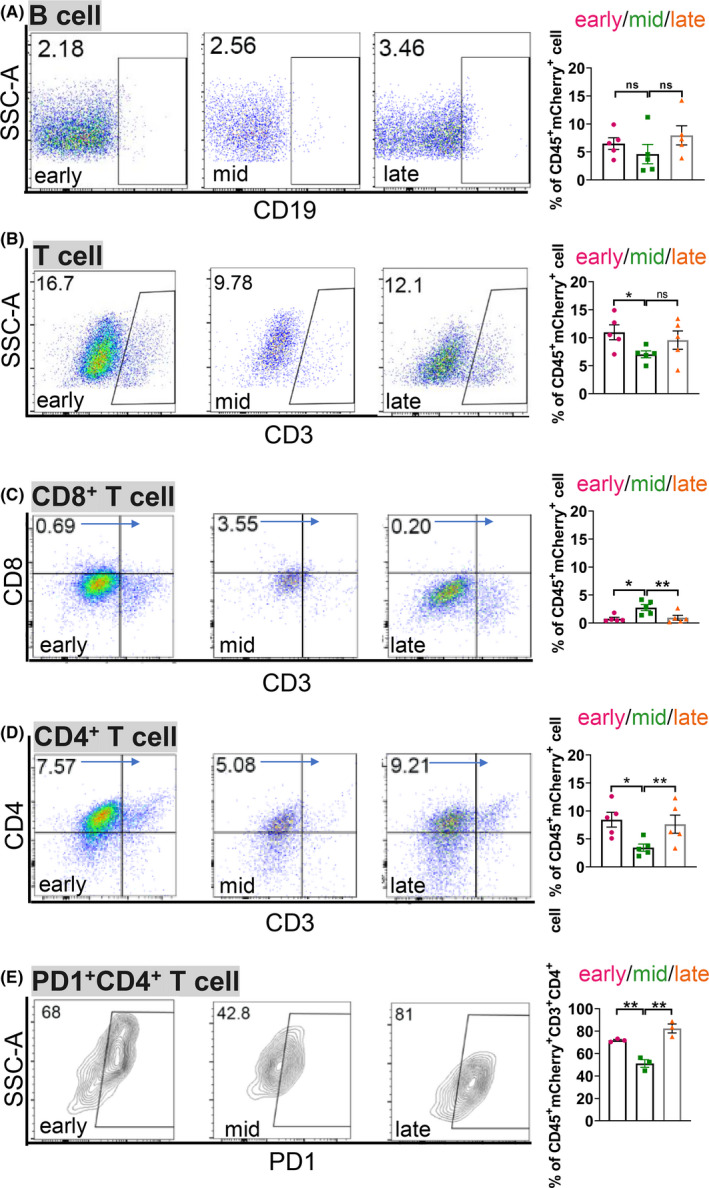
A paucity of CD8^+^ T cells and abundant PD1^+^ CD4^+^ T cells further contribute to the immunesuppressive liver metastatic niche in *rb1^Δ/Δ^p53^Δ/Δ^* ODT. A‐E, Representative flow cytometric analysis and quantification of the ratio of B cells (A), CD3^+^ T cells (B), CD8^+^ T cells (C) and CD4^+^ T cells (D) in CD45^+^ mCherry^+^ cells, and PD1^+^ CD4^+^ T cells (E) in CD45^+^ mCherry^+^ CD4^+^ T cells in *rb1^Δ/Δ^p53^Δ/Δ^* ODT liver metastasis niche at the early, middle, and late stages. Unpaired two‐tailed Student's *t*‐test is used for statistical analysis. The data are presented as the means ± SEMs. *P < .05, **P < .01, n = 5

We further assessed the ratio of mCherry labelled B cells and T cells across different stages of liver metastasis (Supplemental Figure 5C). As shown in Figure 6A, the subset of B cells was rare in mCherry labelled immune cells. It slightly increased from 2.18% at the early stage to 2.56% at the middle stage, and finally reached to 3.46% at the late stage. In contrast, the number of mCherry labelled CD3^+^ T cell was more abundant as compared to B cells. CD3^+^ T cells accounted for around 16.7% of mCherry^+^ immune cells at the early stage, then dropped to 9.78% at middle stage, and finally went up to 12.1% at the late stage (Figure 6B). Of note, the cytotoxic CD8^+^ T cells were very rare in mCherry^+^ T cells, which ranged from 0.20% to 3.55% across different stages (Figure 6C). These results suggested that the rapid progression of liver metastasis was associated with a lack of cytotoxic CD8^+^ T cells in liver metastatic niche. In contrast to CD8^+^ T cells, the ratio of CD4^+^ T cells initially decreased from 7.57% at the early stage to 5.08% at the middle stage. Finally, the ratio of CD4^+^ T cells rebounded to 9.21% at the end stage (Figure 6D). Therefore, there was a negative co‐relation between CD8^+^ T and CD4^+^ T cells across different stages.

We also assessed the expression of immune checkpoint protein PD1 on the surface of T cells.^21^ As shown in Figure 6E, flow cytometric analysis indicated that the PD1^+^ CD4^+^ T cells initially decreased from 68% at the early stage to 42.8% at the middle stage. Intriguingly, the ratio of PD1^+^ CD4^+^ T cells increased to 81% at the late stage. These results suggested that a lack of CD8^+^ T cells and abundance in PD1^+^CD4^+^ T cells further generate an immune‐suppressive liver metastatic niche.

## 4 DISCUSSION

**FIGURE 7:**
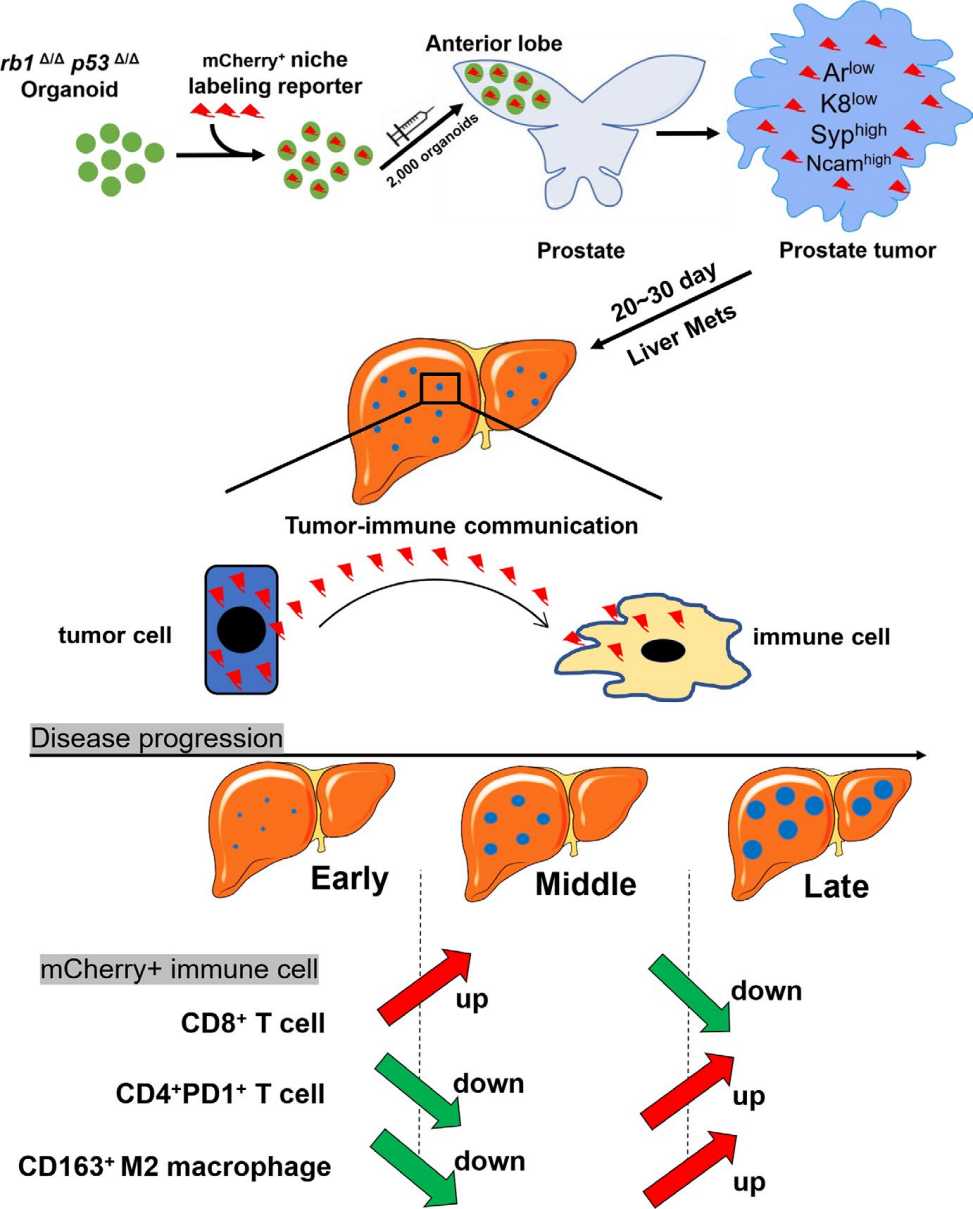
Schematic graph of the dynamic tumor‐immune cell communication in the liver metastatic niche of the *rb1^Δ/Δ^p53^Δ/Δ^* ODT mouse model. A, A schematic model showing a dynamic change of CD8^+^ mCherry^+^ T cells, PD1^+^ CD4^+^ mCherry^+^ T cells, and CD163^+^ M2 macrophages at the early, middle and late stages of liver metastasis in the *rb1^Δ/Δ^p53^Δ/Δ^* ODT model

In the current study, we developed a stable and rapid liver metastasis model of PCa here. Orthotopic inoculation of *rb1^Δ/Δ^p53^Δ/Δ^* tumour organoids in the prostate of WT C57BL/6 mice rapidly developed liver metastasis with high penetrance. *Rb1^Δ/Δ^p53^Δ/Δ^* ODT faithfully recapitulated the histological feature and lineage signature of their parental *rb1^Δ/Δ^p53^Δ/Δ^* GEMM primary tumours. Importantly, the metastatic cells stably sustained neuroendocrine features that were reminiscent of their parental tumour cells in the primary sites. As depicted in Figure 7, using a mCherry‐Luc niche‐labelling reporter, we revealed that the most abundant immune cells in response to tumour cells in liver were macrophages, which were predominantly featured with an M2 phenotype. Dynamic tumour‐immune cell communication was depicted at different stages in the liver metastatic niche. The paucity of CD8^+^ T cells and increased number of PD1^+^ CD4^+^ T cells might contribute to an immune‐suppressive microenvironment that supported the tumour cell colonization in the liver.

In the *rb1^Δ/Δ^p53^Δ/Δ^* ODT model, liver metastases are initiated with a full penetrance as early as 20 ~ 30 days. The traditional approach to generate metastatic PCa GEMM requires time‐ and labour‐consuming way to cross multiple genetically modified mouse lines. In addition, the liver metastasis develops with a low efficiency, takes a long period of time and is highly variable among individual animals. Up to now, there is no ideal *in vivo* model for liver metastasis using patient‐derived cell lines or xenografts to the best of our knowledge. Existing liver metastasis models which use intracardiac injection,^22^ hepatic inoculation^23^ or ectopically splenic implantation^24^ omit the step of tumour cells dissemination from primary prostate sites. In addition, successful transplantation of human PCa cells requires severe immune deficient mice as recipients, which prohibits us from investigation on cancer related immune microenvironment. In contrast, our *rb1^Δ/Δ^p53^Δ/Δ^* ODT model allows a fast and stable generation of liver metastasis in immune sufficient animals.

Previous studies have implied an intrinsic correlation between oncogenic drivers and the propensity of a selective metastatic site in human malignancies. For instance, EGFR^L858R/exon19Δ^ predicts a high propensity of bone and pleura metastases in a group of treating naïve patients with lung adenocarcinoma in late IV stage, while tumour cells with ALK rearrangement are more prone to metastasize to the liver.^25^ In our current work, we carefully compare two frequently used PCa mouse models including *rb1^Δ/Δ^p53^Δ/Δ^* GEMM and *pten^Δ/Δ^p53^Δ/Δ^* GEMM. We find that co‐inactivation of *rb1* and *p53* exhibited liver metastasis with a higher efficiency. However, we do not detect significant liver metastasis in *pten^Δ/Δ^p53^Δ/Δ^* GEMM even at the very end stage of their diseases. A recent literature reported that Rb1 and p53 cooperatively suppress cell lineage plasticity and prevent the transdifferentiation from AR dependent prostate adenocarcinoma to NEPC.^12^ Co‐inactivation of *rb1* and *p53* in the murine prostate confers a significant neuroendocrine feature and resistance to androgen deprivation.^13^ In contrast, *pten* and *p53* mutant PCa display a mixed adenocarcinoma with occasional squamous tumour features. This discrepancy in *rb1^Δ/Δ^p53^Δ/Δ^* GEMM and *pten^Δ/Δ^p53^Δ/Δ^* GEMM models suggest that distinct driver mutations in PCa elite differential molecular programmes and result in different metastasis potential and site preference. Although the cause‐and‐effect relationship remains further investigations, our findings indicated that Rb1 might be a critical gatekeeper that suffices liver metastasis in advanced PCa.

Once spread to the liver, an immune‐suppressive microenvironment is highly required for the survival and growth of metastatic tumour cells in the metastatic niche. Due to the difficulty in collecting clinical liver metastasis specimens and the lack of a reliable liver metastasis mouse model, the tumour‐immune cell communication in the liver metastatic niche remains largely unknown. We introduce a mCherry^+^ niche‐labelling lentiviral reporter system to the *rb1^Δ/Δ^p53^Δ/Δ^* ODT model. Herein, this niche‐labelling reporter has at least uncovered two major interesting findings. *First*, the majority of immune cells that takes up the mCherry protein is CD163^+^ M2 like macrophage. *Second*, insufficient CD8^+^ T cells and abundant PD1^+^ CD4^+^ T cells that react to invading tumour cells might contribute to regional immune suppression in the liver metastatic niche. Interestingly, during the preparation of our manuscript, a very recent paper reported that liver metastases were capable to siphon activated Fas^+^ CD8^+^ T cells from peripheral blood to the liver, where these cytotoxic T cells were eliminated by FasL^+^ CD11b^+^ F4/80^+^ macrophages.^26^ This is in line with our result of an inverse association between the ratio CD8^+^ T cells and M2‐like macrophages across early, middle and late stages in the liver metastatic niche of PCa. Our findings highlight a requirement of an immune‐suppressive environment and reveal the key immune cell component for PCa cell outgrowth in liver metastasis.

Up till now, a few of methods including intracardiac injection^27^ and hemi‐spleen injection^28^ has been introduced to study liver metastasis in different cancer types. However, intracardiac and hemi‐spleen injections fail to mimic the tumour progression in the orthotopic sites where the primary tumour develops and skip the epithelia‐mesenchymal transition (EMT) and intravasation steps during metastasis. Here, we successfully established a stable, rapid and immune sufficient mouse model for PCa liver metastasis, which mimics the entire navigation of tumour metastasis from local site to the liver. Therefore, our model can be used as a powerful tool for to study cellular and molecular mechanisms of liver metastasis. Compared to GEMMs and patient‐derived cell lines or xenografts, it is time‐ and labour‐ friendly and harbours intact immune system. It can serve as a valuable platform for novel therapy development and large‐scale screening of drugs for the treatment of metastatic PCa.

## CONFLICT OF INTEREST

The authors declare no conflicts of interest exist.

## Supporting information

Fig S1

Fig S2

Fig S3

Fig S4

Fig S5

Table S1‐S3

## Data Availability

The data of this study can be obtained from the corresponding authors when requested reasonably.
