# Supplementary figures and images for: A novel mouse model for liver metastasis of prostate cancer reveals dynamic tumour‐immune cell communication
Source: Cell Prolif. 2021 May 21;54(7):e13056. doi: 10.1111/cpr.13056 (PMC8249794; doi:10.1111/cpr.13056)

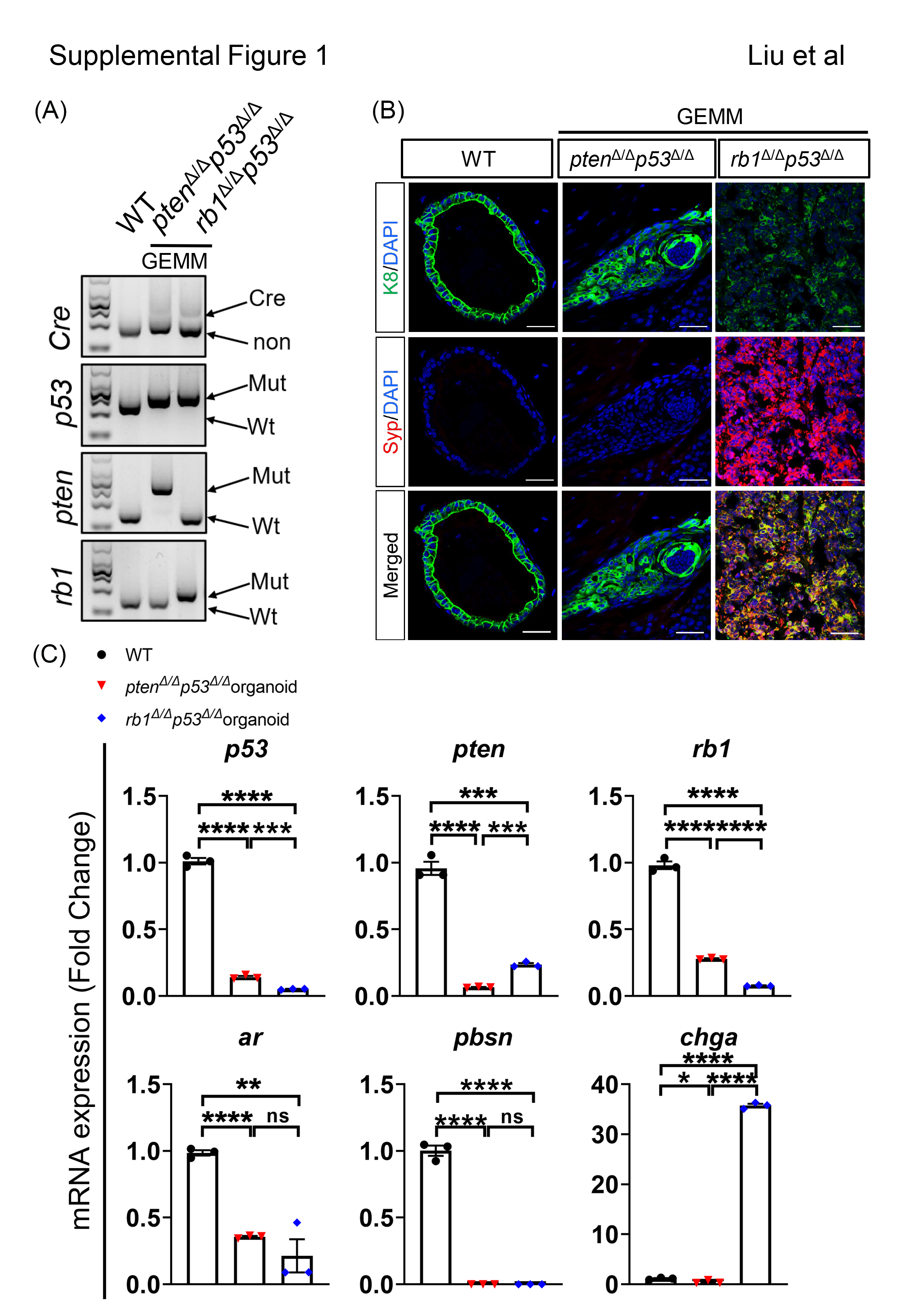

Supplement: Supplementary file 1 — Fig S1 [file CPR-54-e13056-s002.png]

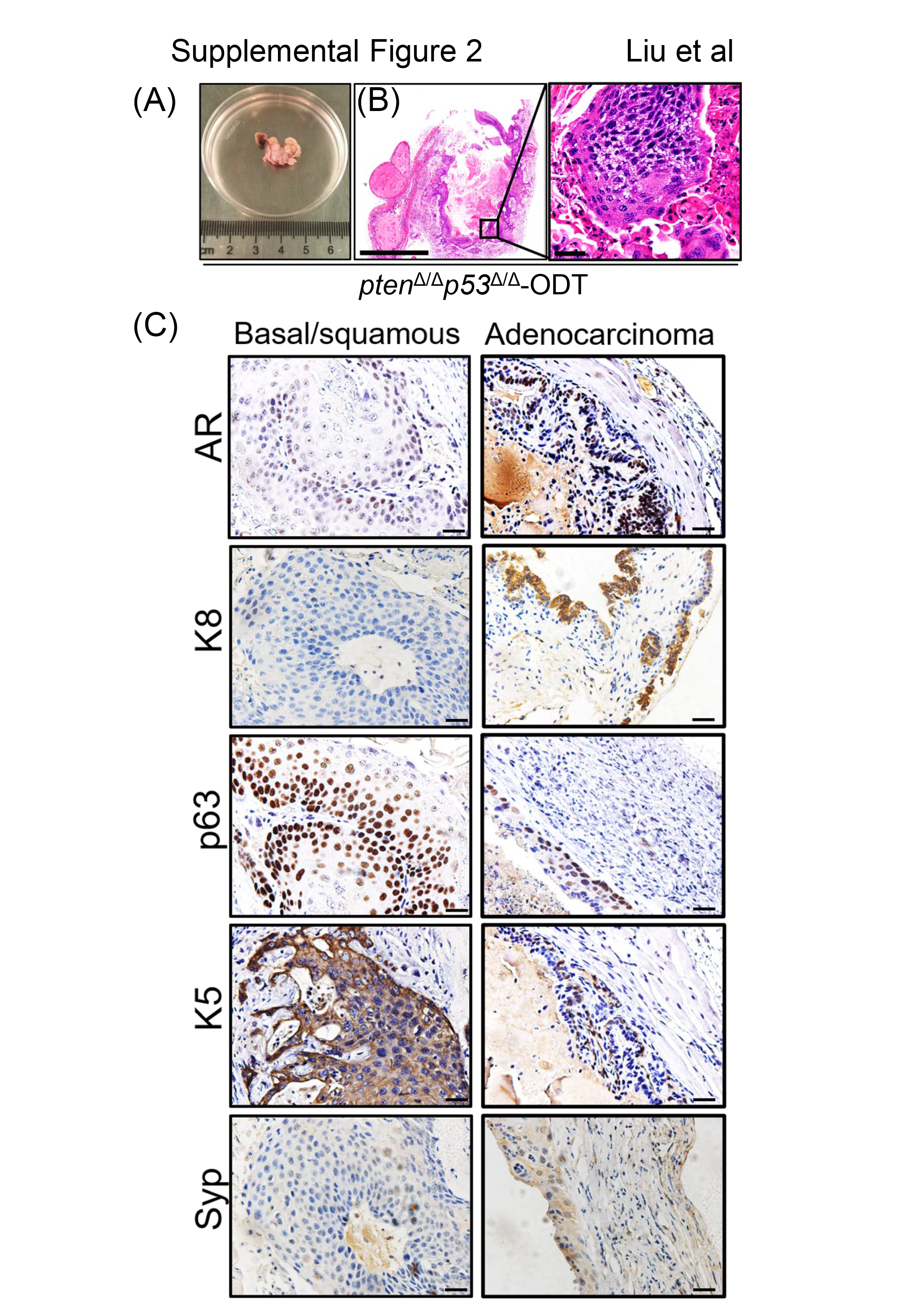

Supplement: Supplementary file 2 — Fig S2 [file CPR-54-e13056-s003.png]

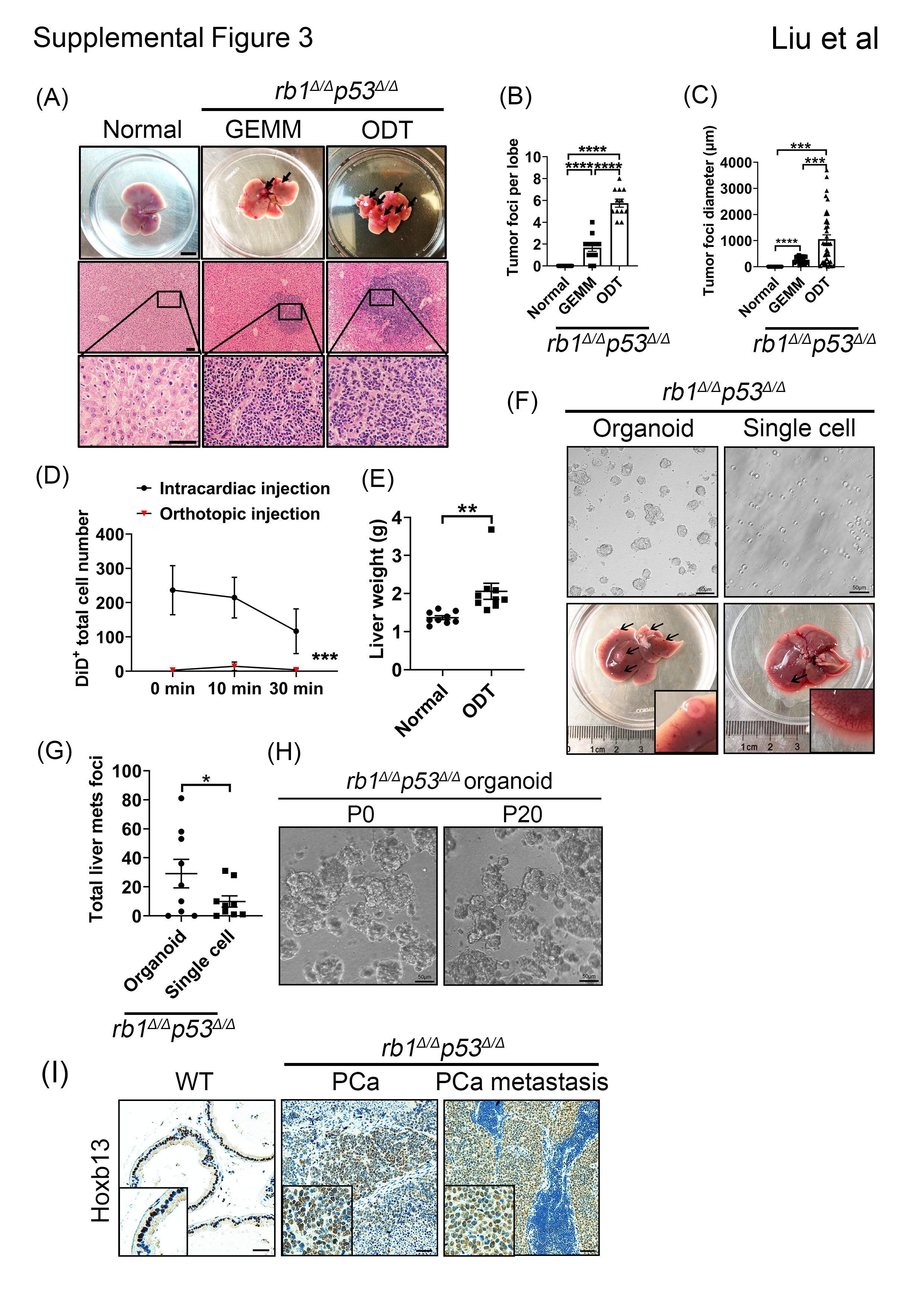

Supplement: Supplementary file 3 — Fig S3 [file CPR-54-e13056-s001.png]

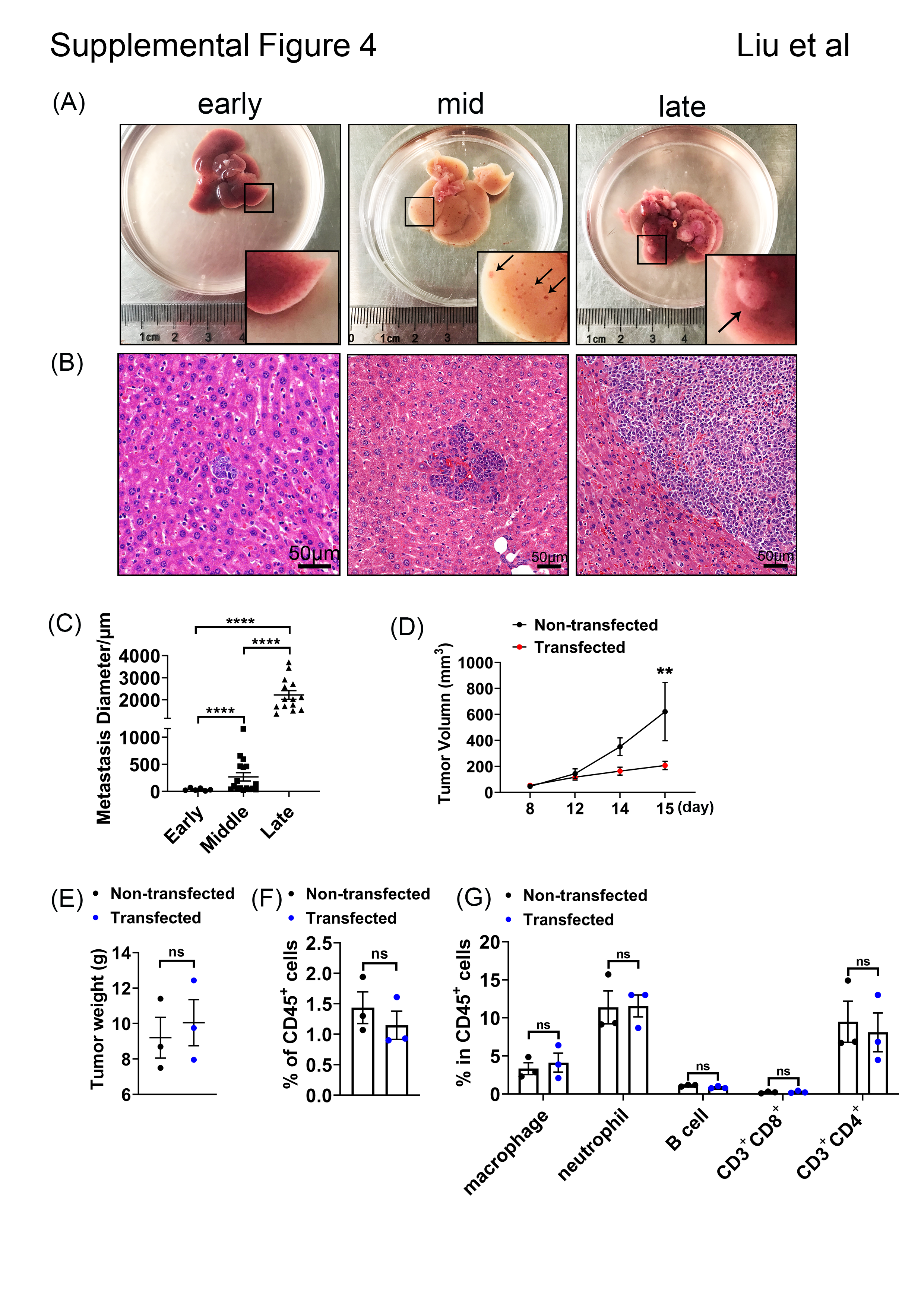

Supplement: Supplementary file 4 — Fig S4 [file CPR-54-e13056-s004.png]

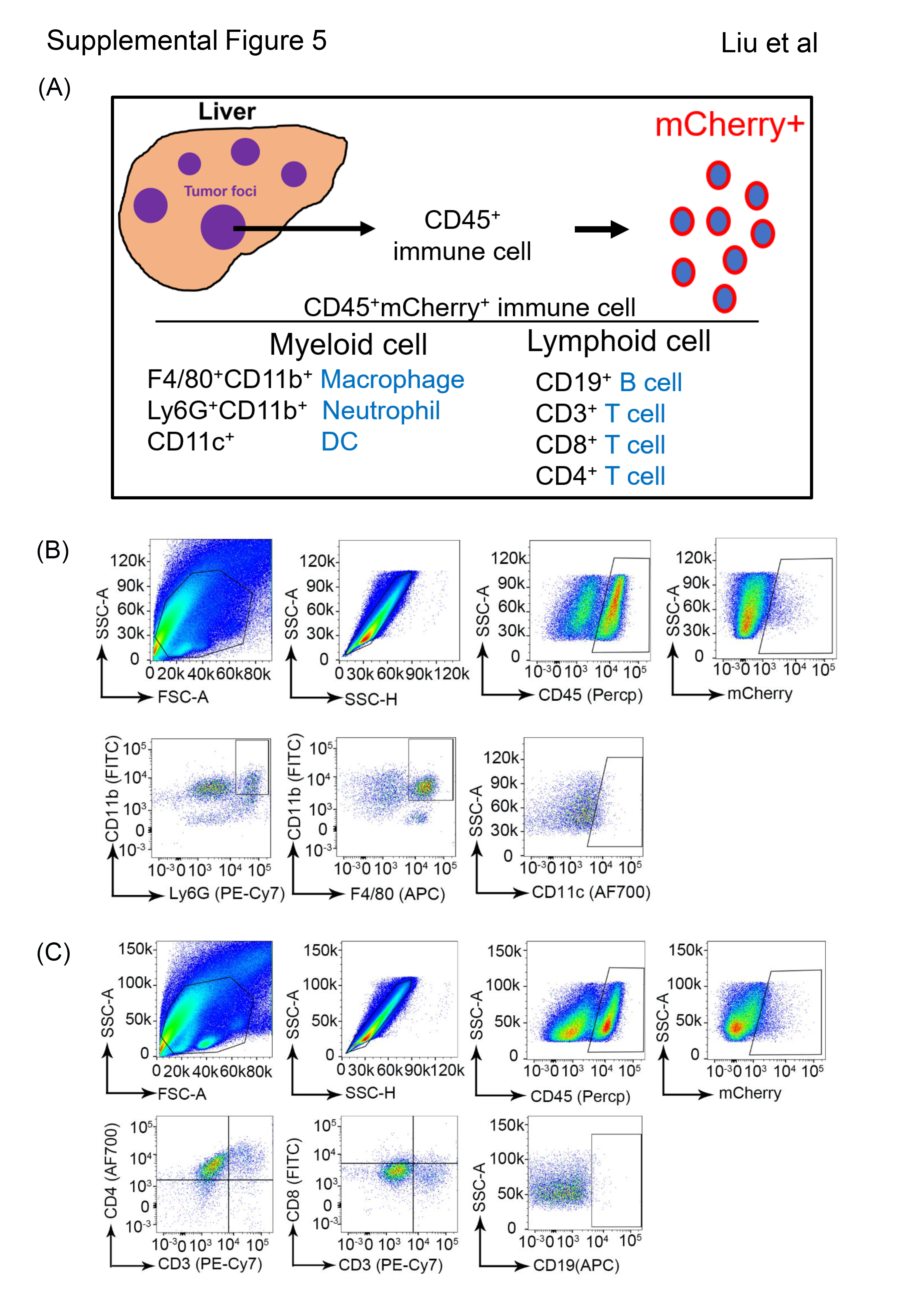

Supplement: Supplementary file 5 — Fig S5 [file CPR-54-e13056-s006.png]
