# Supplementary material for: A novel mouse model for liver metastasis of prostate cancer reveals dynamic tumour‐immune cell communication
Source: Cell Prolif. 2021 May 21;54(7):e13056. doi: 10.1111/cpr.13056 (PMC8249794; doi:10.1111/cpr.13056)
Supplement: Supplementary file 6 — Table S1‐S3 [file CPR-54-e13056-s005.docx]

**Supplemental Methods and Materials**

**2.4 Flow cytometry**

Isolated single cells were first blocked with the CD16/32 antibody for 40 min on ice, then incubated with indicated flow antibodies for 40 min on ice. Cells were washed with PBS and centrifuged at 350 g for 5 min. Flow cytometry analysis or fluorescence activated cell sorting were performed on BD Fortessa or BD Aria II flow cytometer. Antibodies used for flow cytometry were shown in Supplemental Table 2.

**2.5 Organoid culture**

MACS purified EpCAM^+^ epithelial cells were cultured in organoid culture medium consisting of the hepatocyte medium (355056, Corning) with additional components including 10 ng/mL epidermal growth factor (EGF; 355056, Corning), 10 µM Y-27632 (07171, STEMCELL Technologies), Glutamax (35050, Gibco), 5% Matrigel (354234, Corning), 100 nM DHT (8380, Sigma A) and 5% charcoal-stripped FBS (12676, Gibco) to generate organoids. Cell suspension was plated into ultralow-attachment 96-well plates (3474, Corning). Organoids were passaged every 4-5 days at a 1:4 dilution. For serial passage, organoids were washed twice with cold 2% FBS and centrifuged at 350 g for 5 min. Supernatant was discarded and cell pellets were re-suspended in 0.25% trypsin-EDTA for 2 min at 37°C. Single cells were obtained by repeatedly gentle pipetting. Trypsin was neutralized with DMEM containing 10% FBS. Cells were centrifuged at 350 g for 5 minutes, then resuspended with organoid medium and transferred into ultralow-attachment 96-well plates.

**2.6 Organoid specimen sectioning**

To evaluate histopathological characterization and expression levels of proteins of interest, OCT embedding or paraffin embedding of cultured organoids were conducted to make organoid specimen sectioning. Organoids were fixed with 4% PFA at 4°C for 30 min and washed with ice-cold PBS. The fixed organoids were resuspended in 100 μL cold collagen I/setting solution (9:1 v/v) mixture. Setting solution was prepared by mixing 1mM NaOH and 10×RPMI-1640 at a ratio of 2:1. The organoid and collagen mixture was left on a petri dish and solidified at 37°C for 2h. Then the solidified mixture was fixed in 4% PFA (for OCT embedding) or 10% neutral buffered formalin (for paraffin embedding). After the embedding block was done, the specimen was cut with microtomes at a thickness of 4 μm per section.

**2.7 Lentivirus packaging and titer assessment**

Lentivirus was produced by 293T cells transfected by the virus production system containing lentiviral plasmid, viral packaging vector (psPAX2) and viral envelope vector (pMD2G) at a 3:2:1 DNA ratio. DNA transfection were conducted using PEI (23966-2, Polysciences). The sLP–mCherry-luciferase sequence was cloned into a plentiviral backbone. Viral supernatant was harvested at 48 hours post-transfection. For virus titer assessment, 75,000 293T cells were seeded into each well of a 6-well dish and incubated overnight. Lentivirus and polybrene (10μL/mL) were added into cell culture medium for infection for 48 h. Positively infected cells were numerated by flow cytometry and lentivirus titer was calculated accordingly.

**2.8 Lentiviral infection of prostate epithelial cells**

Ultracentrifuge lentiviral supernatant collected from 293T cells in an Optima ultracentrifuge from Beckman Coulter Life Sciences with SW 32 Ti rotor using density gradient method for virus concentration. 3 mL 20% sucrose was added to the bottom of the ultracentrifuge tube. Ultracentrifuge was performed at 27,000 rpm for 2 h at 4°C. For prostate epithelial cell infection, organoids were dissociated into single cells and then subjected to spin inoculation for lentiviral transfection. Briefly, the mixture of cell suspension and virus in 6-well plates was centrifuged at 3,000 rpm for 3h at room temperature (RT). The transfected cells were plated into ultralow-attachment 96-well plates. 48 h later, the percent of mCherry^+^ cells was numerated using flow cytometry.

**2.9 Intracardiac injection**

DiD^+^ fluorescent labeled *rb1^Δ/Δ^p53^Δ/Δ^* organoids were dissociated into single cells with 0.25% trypsin-EDTA at 37°C for 2 min. After centrifuging at 350 g for 5 minutes, cells were resuspended with PBS. 40 μm cell strainer was then used to isolate single cells. Mice were anesthetized using continuous flow of 1% isoflurane. 100 µL of single cell suspension was injected into the left ventricular cavity. Surgery was performed on a heating pad until mice completely recovered from anesthesia.

**2.10 Immunofluorescent, immunohistochemical and H&E staining**

**For immunofluorescent staining,** murine prostates were fixed in 4% PFA overnight and then dehydrated in 30% sucrose for additional 24 hours. The tissue was embedded in OCT compound and frozen in −80 °C refrigerator for 30 min. Frozen sections were cut at a thickness of 6 µm. Sections were performed heat-induced antigen retrieval with 0.01 M Citrate Antigen Retrieval Solution (PH 6.0). Sections were then blocked in 10% donkey serum for 1 hour at RT. Primary antibodies were diluted in 1% donkey serum with 0.3% Triton X-100 in PBS and were applied to sections at 4°C overnight. The slides were then washed 3 times with PBS and incubated with secondary antibodies at RT for 1 hour. Slides were then washed 3 times with PBS and mounted using Vector Shield Mounting Medium with DAPI (H-1200, Vector). Image acquisition was performed on Zeiss 710 Confocal Microscope at a 400 magnification. Antibodies used in the experiment were listed in Supplementary Table 2.

**For immunohistochemical staining,** paraffin-embedded prostate tissues were cut into 5-μm-thick sections, and then deparaffinized in xylol and rehydrated in ethanol. Slides were stained with indicated primary antibody as described above. For the final step, slides were incubated with horseradish peroxidase conjugated secondary antibodies at RT for 1 hour. The slides were then applied with the GT Vision III Immunohistochemistry Detection Kit (Gene Tech Inc., Shanghai, China) according to the manufacturer’s instructions and were counterstained with Hematoxylin.

**For H&E staining,** paraffin-embedded prostate tissue slides were deparaffinized and rehydrated as stated in above immunohistochemistry experiment. Hematoxylin was applied to slides for 15 min at RT. The slides were washed with ddH_2_O for 5 min. 0.5% hydrochloric acid in 70% reagent grade alcohol was used for 10 s to greatly improve reproducibility of staining results and help achieve standardization. The slides were then washed with ddH_2_O for 5 min, stained with eosin for 30 s, and washed with 75% ethanol. Slides were mounted using neutral balsam.

**2.11 RNA Extraction, Reverse Transcription, and Quantitative Reverse Transcription PCR**

Total RNA was extracted using TRIzol (15596026, Invitrogen). Reverse transcription of RNA to cDNA was performed using the PrimeScript RT Reagent Kit (Takara, RR037A). qPCR was conducted using SYBR Premix Ex Taq (Takara, RR420A) according to manufacturer’s protocol. For relative quantification by qPCR, *gapdh* was used as an internal reference gene. Primers in qPCR were listed in Supplemental Table 3.

TABLE. S1 Primer sequences of genotyping

| Primer ID | Sequence (5’ to 3’) | Primer type |
| --- | --- | --- |
| Pbsn-cre | CTGAAGAATGGGACAGGCATTG | Transgene Forward |
|  | CATCACTCGTTGCATCGACC | Transgene Reverse |
|  | CAAATGTTGCTTGTCTGGTG | Internal Positive Control Forward |
|  | GTCAGTCGAGTGCACAGTTT | Internal Positive Control Reverse |
| Pten | CAAGCACTCTGCGAACTGAG | Forward |
|  | AAGTTTTTGAAGGCAAGATGC | Reverse |
| Rb1 | CTCATGGACTAGGTTAAGTTGTGG | Forward |
|  | GCATTTAATTGTCCCCTAATCC | Reverse |
| p53 | GGTTAAACCCAGCTTGACCA | Forward |
|  | GGAGGCAGAGACAGTTGGAG | Reverse |

TABLE. S2 Antibody list

| Application | Target antigen | Target species | Conjugation | Dilution | Vendor | Catalog # |
| --- | --- | --- | --- | --- | --- | --- |
| Flow Cytometry | CD11b | Mouse | FITC | 1:100 | eBioscience | 11-0112-85 |
|  | CD25 | Mouse | APC | 1:100 | eBioscience | 17-0251-81 |
|  | CD326 | Mouse | Biotin | 1:100 | eBioscience | 13-5791-80 |
|  | CD8a | Mouse | FITC | 1:100 | eBioscience | 11-0081-82 |
|  | F4/80 | Mouse | APC | 1:100 | eBioscience | 17-4801-80 |
|  | CD45 | Mouse | Percp | 1:100 | BioLegend | 103130 |
|  | CD11c | Mouse | AF700 | 1:100 | BioLegend | 117320 |
|  | CD4 | Mouse | AF700 | 1:100 | BioLegend | 100430 |
|  | CD31 | Mouse | APC | 1:100 | BioLegend | 102410 |
|  | CD19 | Mouse | APC | 1:100 | BioLegend | 115512 |
|  | Ly-6G | Mouse | PE-Cy7 | 1:100 | BioLegend | 127618 |
|  | CD163 | Mouse | PE-Cy7 | 1:100 | eBioscience | 25-1631-82 |
|  | Ly-6C | Mouse | PE-Cy7 | 1:100 | eBioscience | 25-5932-82 |
|  | FOXP3 | Mouse | FITC | 1:100 | eBioscience | 53-4776-42 |
|  | CD86 | Mouse | AF700 | 1:100 | eBioscience | 56-0862-82 |
|  | CD3 | Mouse | PE-Cy7 | 1:100 | BD | 560591 |
|  | alpha-Fetoprotein- | Mouse | AF700 | 1:100 | Novus | NBP2-48016AF700 |
|  | alpha-SMA- | Mouse | AF488 | 1:100 | Novus | NBP2-44464AF488 |
| Magnetic Cell Sorting | MicroBeads | Mouse | Biotin | 20 µL per 10^7^ total cells | Miltenyi | 130-090-485 |
| IF and IHC | Fibronectin | Mouse | Unconjugated | 1:100 | Abcam | ab2413 |
|  | Collagen I | Mouse | Unconjugated | 1:100 | Abcam | ab34710 |
|  | α-SMA | Mouse | Unconjugated | 1:100 | Santa Cruz | sc-53142 |
|  | Androgen Receptor | Mouse | Unconjugated | 1:100 | Abcam | ab133273 |
|  | p63 | Mouse | Unconjugated | 1:100 | Abcam | ab735 |
|  | Krt8 | Mouse | Unconjugated | 1:100 | Abcam | ab53280 |
|  | Synaptophysin | Mouse | Unconjugated | 1:100 | BD | 611880 |
|  | Albumin | Mouse | Unconjugated | 1:100 | CST | 4929 |
|  | Ki-67 | Mouse | Unconjugated | 1:100 | Abcam | Ab15580 |
|  | Ncam1 | Mouse | Unconjugated | 1:100 | CST | 99746S |
|  | IgG | Mouse | AF 488 | 1:500 | Invitrogen | A-21208 |
|  | IgG | Mouse | AF 594 | 1:500 | Invitrogen | A-21207 |

TABLE. S3 Primers sequence of qPCR

| Gene | Forward (5’ to 3’) | Rerverse (5’ to 3’) |
| --- | --- | --- |
| Ar | CTGGGAAGGGTCTACCCAC | GGTGCTATGTTAGCGGCCTC |
| Chga | ATCCTCTCTATCCTGCGACAC | GGGCTCTGGTTCTCAAACACT |
| Pbsn | TGTCACACGAGTGGCTGGAGTT | CCGTGTCCATGATACGCTGTAC |
| Pten | CCTTTTGAAGACCATAACCCACC | GAATTGCTGCAACATGATTGTCA |
| Rb1 | TCGATACCAGTACCAAGGTTGA | ACACGTCCGTTCTAATTTGCTG |
| p53 | CTCTCCCCCGCAAAAGAAAAA | CGGAACATCTCGAAGCGTTTA |
